# Observations on Eye Conditions Met with in Malta, 1916-17, Occurring among British Troops in the Balkans and Malta Garrison

**Published:** 1919

**Authors:** 


**Observations on Eye Conditions Met with in Malta, 1916-17, Occurring among British Troops in the Balkans and Malta Garrison.** By E. M. Maxwell. *The British Journal of Ophthalmology,* October, 1918.

Maxwell mentions the divergence of opinion encountered among observers of the pathology of sunlight, and gives an account of the ocular lesions seen among the British troops in the Balkans and in Malta. These lesions affected: 1) *The Conjunctiva.* Cases of persistent hyperemia, cases with chronic enlargement of the lymph follicles, and 3 cases of pterygia, apparently due to irritated pinguеculae, were recorded. 2) *The Cornea.* Cases of superficial and relapsing keratitis due to sunlight and dust. 3) *The Retina.* Cases of night blindness unaccompanied by fundus defect or any general disease, and one case from the Balkan front, with fundus defects, probably caused by exposure to sunlight.

Further, the author speaks of the unsuitability of albinos and high myopes with thinning of the choroid and scanty amount of retinal pigment, for service in the Near East, in connection with the protective power of the body pigment against the effects of sunlight; he also insists upon the importance of protective glasses needed chiefly in air work and lookout posts near wide stretches of water, and gives the most efficacious types to be used: airmen's goggles, cutting off actinic rays; dark glasses, to be served out in cases of chronic blepharitis, irritable conjunctivae, etc., although their use in the front line must be limited; Crookes' " A " glass which cuts off 27 o/o of the thermic rays and all the actinic rays.

A survey of a large number of refraction cases was made by the author, who then drew the following conclusions:

1) No visual defect, resulting from a refractive error, should cause exemption from military service, and cases of defective vision due to pathological conditions must be dealt with individually.

2) The prescribing of glasses for men with refractive errors should be avoided, except in cases of medium degree myopes, as a prophylaxis against progression in cases of men required for clerical work or for posts demanding marked acuity of vision.

The prescribing of glasses at the front for night blindness, caused or accentuated, according to many observers, by errors of refraction, cannot be regarded as a practical measure.

In the case of wounded or sick men suffering at the same time from refractive errors, glasses should be prescribed in order to avert headaches and general discomfort. Their use may be discontinued on return of health.

3) Ambliopia, the result of squint or refractive error, should not cause exemption from active service, as men with amblyopic eyes usually retain good peripheral vision; moreover, they have been accustomed to their condition from early life.

Where the loss of sight of one eye has been sustained recently, the man is unfit for active service, owing to lessened effectiveness.

